# Identification and recombinant expression of anandamide hydrolyzing enzyme from *Dictyostelium discoideum*

**DOI:** 10.1186/1471-2180-12-124

**Published:** 2012-06-25

**Authors:** Dhamodharan Neelamegan, Ian C Schoenhofen, James C Richards, Andrew D Cox

**Affiliations:** 1 National Research Council, Institute for Biological Sciences, 100, Sussex Drive, Ottawa, ON, K1A 0R6, Canada

## Abstract

**Background:**

Anandamide (Arachidonoyl ethanolamide) is a potent bioactive lipid studied extensively in humans, which regulates several neurobehavioral processes including pain, feeding and memory. Bioactivity is terminated when hydrolyzed into free arachidonic acid and ethanolamine by the enzyme fatty acid amide hydrolase (FAAH). In this study we report the identification of a FAAH homolog from *Dictyostelium discoideum* and its function to hydrolyze anandamide.

**Results:**

A putative FAAH DNA sequence coding for a conserved amidase signature motif was identified in the Dictyostelium genome database and the corresponding cDNA was isolated and expressed as an epitope tagged fusion protein in either *E.coli* or Dictyostelium. Wild type Dictyostelium cells express FAAH throughout their development life cycle and the protein was found to be predominantly membrane associated. Production of recombinant HIS tagged FAAH protein was not supported in *E.coli* host, but homologous Dictyostelium host was able to produce the same successfully. Recombinant FAAH protein isolated from Dictyostelium was shown to hydrolyze anandamide and related synthetic fatty acid amide substrates.

**Conclusions:**

This study describes the first identification and characterisation of an anandamide hydrolyzing enzyme from *Dictyostelium discoideum*, suggesting the potential of Dictyostelium as a simple eukaryotic model system for studying mechanisms of action of any FAAH inhibitors as drug targets.

## Background

Anandamide [1] is a mammalian endogenous lipid that binds cannabinoid receptors which are mainly present in the central nervous system and immune cells. Anandamide was identified in 1992 and named after the Sanskrit word ananda, meaning bliss or delight. Anandamide acts as an agonist for the central cannabinoid receptor (CB1) and is therefore referred to as cannabinoid. It mimics pharmacological effects of Δ^9^tetrahydrocannabinol, an active ingredient of marijuana [2]. Action of anandamide is terminated by the enzyme fatty acid amide hydrolase (FAAH) [3]. FAAH was originally identified in 1996 from rat liver plasma membrane and later FAAH homologs were identified from other sources including human, porcine, and Arabidopsis. FAAH belongs to a large group of proteins containing a conserved amidase signature motif [4,5]. FAAH can also hydrolyze, in addition to anandamide, other fatty acid derivatives like *N*-oleoylethanolamine and *N*-palmitoylethanolamine collectively referred as *N*-acylethanolamines (NAEs) [6]. Studies on mammalian FAAH have provided more information on NAEs role in regulating various physiological functions like sleep and pain [7-9]. Recent studies on NAEs reveal further biological roles in appetite suppression, vasodilatation, cardiac function and inflammation [10-12]. Therefore any FAAH inhibitors which intervene in NAE’s bioactivity promise to be a novel class of therapeutics and much drug discovery research is being actively pursued in this regard [13,14]. Anandamide is yet to be found in Dictyostelium, but its precursor *N*-acylphosphatidylethanolamine (NAPE) has previously been identified [15]. In mammalian cells anandamide is believed to originate from hydrolysis of NAPE by phospholipase D (PLD). In Dictyostelium, a PLD homolog PldB was identified and proposed to have a similar function [16]. Identification of FAAH suggests that regulation of NAE signalling could occur in Dictyostelium and thus Dictyostelium could be utilised as a simple eukaryotic model to study NAE functions in parallel with mammalian systems. Dictyostelium has been used to study cell motility, chemotaxis, cell differentiation and morphogenesis enabling significant contributions to an understanding of similar processes in mammalian systems. Recently Dictyostelium has been used as a system to study the mechanism of action for mood stabilizing drugs like Lithium and Valproic acid [17]. Therefore the identification of any new drug target enzyme such as FAAH or any drug processing mechanisms in Dictyostelium suggests further potential for the use of Dictyostelium in human biomedical research. Dictyostelium offers an attractive system to study such processes by gene manipulation studies because of its small 34 Mbp haploid genome harbouring many homologous genes found in higher eukaryotes [18].

## Results

### Amino acid sequence analysis

**Figure 1:**
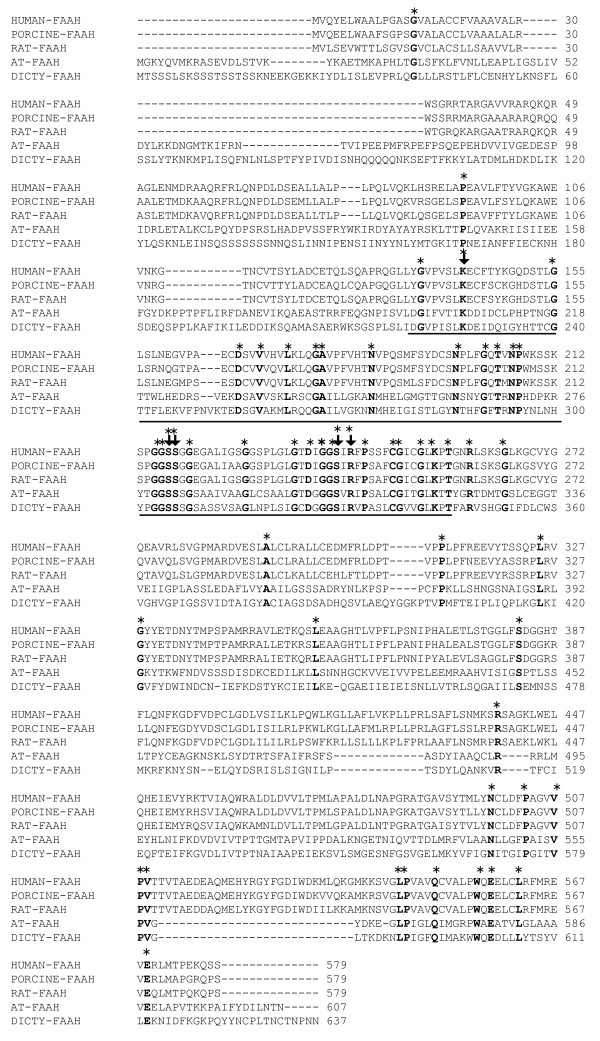
**Comparative alignment of amino acid sequences of Dictyostelium FAAH with mammalian and Arabidopsis FAAH.** Full length amino acid sequence alignment of human [NCBI:NP_001432], porcine [NCBI:NP_999079], rat [NCBI:NP_077046], Arabidopsis (AT) [NCBI:AAP83139] and Dictyostelium (Dicty) [NCBI:XP_643382]. The amidase signature (AS) sequence is underlined and consists of about 126 amino acids. Asterisks denote identical amino acids and residues essential for FAAH activity in rat is indicated by arrow mark.

A putative FAAH in Dictyostelium was identified by a bioinformatics approach searching for a human FAAH homolog in the Dictyostelium genome. Dictyostelium DNA sequence *DDB_G0275967* (http://dictybase.org/gene) [GenBank: XM_638290] containing coding sequences for characteristic amidase signature motifs [19] was identified and found to be located on chromosome 2 in the annotated Dictyostelium genome data base. [GenBank: XM_638290] will be referred to as Dictyostelium FAAH as the protein’s amino acid sequence analysis and other experimental results confirm its function to be similar to mammalian FAAH. The calculated molecular weight of Dictyostelium FAAH is 70 kDa and domain architecture analysis (http://www.ncbi.nlm.nih.gov/structure/cdd) reveals the presence of an amidase domain composed of a characteristic amidase signature (AS) sequence (Figure 1). The consensus amidase signature sequence has a conserved GSS(G/A/S)G (residues 304 to 308) motif shared among many proteins in the amidase class including glutamyl-t-RNA amidotransferase subunit A of *Methanococcus jannaschii* and FAAH from human, porcine, rat, Arabidopsis and Dictyostelium. FAAH from human, porcine and rat are composed of 579 amino acids and FAAH from Dictyostelium and Arabidopsis contain 637 and 607 amino acids, respectively. FAAH full length protein amino acid sequence from Dictyostelium lacks significant identity when compared to FAAH from human (20%), porcine (20%), rat (20%), and Arabidopsis (32%) (Figure 1), but identity across the amidase signature sequence increased to 40%, 38%, 38%, and 50%, for the human, procine, rat, and Arabidopsis FAAH homologs. The serine residues at 217 and 241 found to be essential for rat FAAH activity [20] were also conserved in AS sequence of Dictyostelium FAAH. Other catalytically important residues Lys142, Ser218 and Arg243 found in rat were also conserved in Dictyostelium.

### Recombinant enzyme expression and affinity purification of FAAH in Dictyostelium and *E. coli*

**Figure 2:**
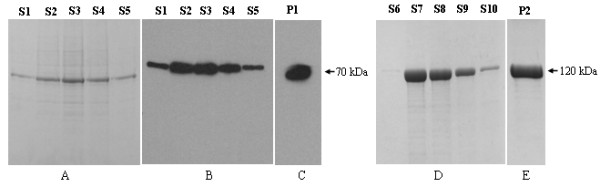
**(A) Coomassie staining of purified HIS-FAAH recombinant protein from Dictyostelium.** Dictyostelium cells AX3FAAH expressing HIS-FAAH were lysed and the recombinant protein was bound to Ni-NTA resin. Resin bound protein was eluted using lysis buffer containing 200 mM Imidazole and the eluate fractions S1, S2, S3, S4, S5 were resolved on 10% SDS-PAGE and Coomassie stained. (**B**) Western blotting analysis. Fractions analysed in Figure 2A were analysed by Western blotting using anti-HIS antibody. (**C**) Western blotting analysis. Fractions analysed in Figure 2A/2B were pooled together (P1) and analysed by Western blotting using anti-FAAH polyclonal antibody and the same fraction was used in enzyme kinetic assay. (**D**) Coomassie staining analysis of purified recombinant MBP-FAAH protein from *E.coli*. Cells expressing recombinant MBP-FAAH were lysed and the recombinant protein was bound to amylose resin. Resin bound recombinant protein was eluted using lysis buffer containing 15 mM maltose and the eluate fractions S6, S7, S8, S9, S10 were resolved on 10% SDS-PAGE and Coomassie stained. (**E**) Coomassie staining analysis. Fractions analysed in Figure 2D were pooled together (P2) and analysed by Coomassie staining.

FAAH was expressed in Dictyostelium as an N-terminal HIS tag fusion protein. FAAH was found to be predominantly a membrane associated protein and to improve yield of the purified protein, a 0.1% concentration of Triton X-100 was used in lysis buffer to solubilise membrane fractions. Cells expressing recombinant HIS-FAAH protein (AX3FAAH) were solubilised in lysis buffer and subjected to Ni-NTA affinity chromatography separation. Purified protein obtained was analyzed by Coomassie staining (Figure 2A) and Western blotting analysis (Figure 2B, C) using anti-HIS antibody (Sigma-Aldrich, Oakville, ON, Canada) and anti-FAAH polyclonal antibody (as described in materials and methods) respectively. Initial attempts to express FAAH as a HIS tag fusion protein in *E.coli* were not successful, as both N-terminal HIS and C-terminal HIS fusions to FAAH were unstable and only a small amount of the protein was made and this was only found in inclusion bodies. Alternatively, in order to simplify large scale recombinant protein production, FAAH was expressed and purified as a recombinant maltose binding protein (MBP) fusion protein from *E.coli* (Figure 2D, E). Recombinant FAAH when expressed as N-terminal MBP fusion protein (MBP-FAAH) in *E.coli* produced a higher yield of soluble recombinant protein. Recombinant FAAH when produced in either Dictyostelium or *E.coli* migrated on SDS-polyacrylamide gels, consistent with no significant post-translation modification.

### Functional identification of Dictyostelium FAAH

**Figure 3:**
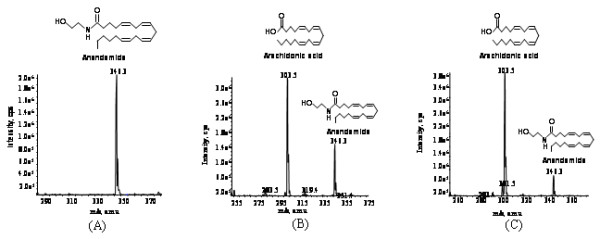
**CE-ES-MS analysis of anandamide hydrolysis by recombinant FAAH from both Dictyostelium and*E.coli.*** (**A**) CE-ES-MS analysis of control reaction having anandamide alone in the reaction buffer without enzyme was analyzed. Negative ion mode product ion scan of mass [*m/z* 346.3]^-^ corresponds to substrate anandamide. Inset figure is the structure of anandamide. (**B**) CE-ES-MS analysis of anandamide hydrolysis by recombinant HIS-FAAH purified from Dictyostelium. Negative ion mode product ion scan of mass [*m/z* 346.3]^-^ corresponds to substrate anandamide and mass [*m/z* 303.5]^-^ corresponds to hydrolyzed product arachidonic acid. Inset figures are the structure of anandamide and arachidonic acid. (**C**) CE-ES-MS analysis of anandamide hydrolysis of recombinant MBP-FAAH purified form *E.coli*. Negative ion mode product ion scan of mass [m/z 346.3]^-^ corresponds to substrate anandamide and mass [m/z 303.5]^-^ corresponds to hydrolyzed product arachidonic acid. Inset figures are the structure of anandamide and arachidonic acid.

Dictyostelium recombinant FAAH expressed as N-terminal HIS tagged fusion protein (HIS-FAAH) in Dictyostelium and as N-terminal MBP tagged fusion protein (MBP-FAAH) in *E.coli* hydrolyzed anandamide to free arachidonic acid and ethanolamine as determined by CE-ES-MS (Figure 3A, B, C). Dictyostelium FAAH was also capable of hydrolyzing synthetic *p*-nitroanilide substrates arachidonoyl *p*-nitroaniline (A*p*NA) and decanoyl *p*-nitroaniline (D*p*NA) which were further used in kinetics studies.

### Catalytic properties

**Figure 4:**
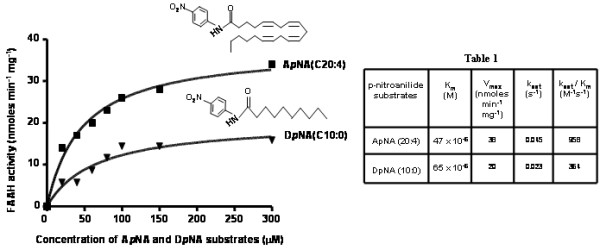
**Kinetic characterization of affinity purified recombinant HIS-FAAH from Dictyostelium.** Initial velocity measurements were made at increasing concentration of arachidonoyl *p-*nitroaniline (A*p*NA) and decanoyl *p*-nitroaniline (D*p*NA) substrates. Reaction was initiated by addition of 10μg of HIS-FAAH protein purified from Dictyostelium and the reaction was incubated at 37°C for 30 min. Data points are mean ± S.D. values of specific activity from triplicate assays from single batch of enzyme purification and plots were generated by fitting the data points into Michaelis-Menten equation using prism software version 3.0. Inset figures are the structures of A*p*NA and D*p*NA**.** Inset Table 1 details kinetic parameters of HIS-FAAH isolated from Dictyostelium were estimated by fitting the data in Figure 4, to Michaelis-Menten equation.

**Figure 5:**
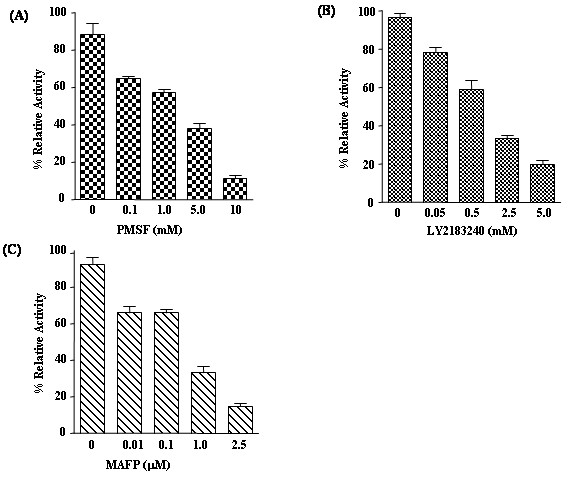
**Effect of different mechanism based inhibitors (A) PMSF, (B) LY2183240 and (C) MAFP on Dictyostelium FAAH activity.** 10μg of HIS-FAAH protein purified from Dictyostelium were incubated for 30 min at 37°C with 100μM arachidonoyl *p*- nitroaniline substrate in the absence (0 mM) or presence of increasing concentration of PMSF, LY2183240 and MAFP. Calculated specific activity of the enzyme reactions with and without the inhibitors were represented as % relative activity. The data are means ± S.D. of three replicate experiments.

Recombinant HIS-FAAH purified from Dictyostelium was analyzed for fatty acid amide hydrolase activity by measuring the rate of hydrolysis of *p*-nitroanilide substrates A*p*NA (C20:4) and D*p*NA (C10:0) (Figure 4), which were previously used to characterize binding and catalytic specificities of mammalian FAAH enzymes [21]. Dictyostelium FAAH exhibited Michaelis-Menten kinetics on these substrates. Specific constant *k*_cat_/*K*_m_ values (Table one- inset in Figure 4) observed for A*p*NA having long chain unsaturated fatty acid (C20:4) were slightly higher when compared to D*p*NA (C10:0), which may indicate the enzyme’s preference for longer unsaturated acyl chains. Similar observations were made with mammalian FAAH where the enzyme showed a 10 fold preference for anandamide versus *N*-palmitoylethanolamine [22]. The *k*_cat_ values of HIS-FAAH towards A*p*NA and D*p*NA when compared with rat FAAH were about 10 and 24 times less, respectively. Purified recombinant FAAH enzymes from both Dictyostelium and *E.coli* exhibited pH optima at 9.0 which were similar to the mammalian FAAH enzymes characterized to have a pH optimum from 9 to 10. Compounds that inhibit enzymatic activity via different mechanisms, phenylmethylsulfonyl fluoride (PMSF), LY2183240 and methyl arachidonoyl fluorophosphonate (MAFP) were tested on Dictyostelium FAAH in order to monitor changes in activity. Non-specific irreversible serine protease inhibitor PMSF was modestly effective and inhibited about 58% at 5 mM (Figure 5A). LY2183240, a serine hydrolase inhibitor which inactivates FAAH by covalent binding, was also modestly effective against Dictyostelium FAAH and inhibited about 62% at 2.5 mM (Figure 5B). Irreversible active site targeted inhibitor MAFP had potent inhibition against Dictyostelium FAAH and inhibited about 63% at 1.0μM (Figure 5C).

### Identification of FAAH in Dictyostelium

**Figure 6:**
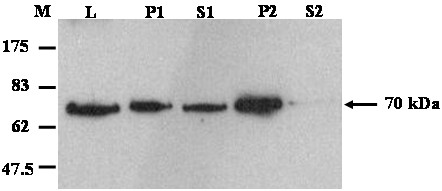
**Western blotting analysis of distribution of HIS-FAAH in membrane fractions of Dictyostelium.** Total cellular protein (L) from AX3FAAH cells were fractionated into 13,000*xg* membrane and cytosol fractions (P1 and S1 respectively) and 100,000*xg* membrane and cytosolic fractions (P2 and S2 respectively). Described membrane and cytosolic fractions were separated on 10% SDS-PAGE and subjected to Western blotting using anti-HIS antibody. M represents molecular mass standard in kDa.

The production of FAAH protein in Dictyostelium was confirmed at the protein level. Dictyostelium anti-FAAH polyclonal antibodies raised in rabbits (as described in materials and methods) were used to detect FAAH production during Dictyostelium development. To trace the *in vivo* FAAH protein production profile, wild type Dictyostelium cells allowed to develop on phosphate agar plates at different stages of development from independent single cell stage through multi-cellular fruiting body, were harvested. Total proteins isolated from the harvested cells were analyzed for FAAH expression by Western blotting using anti FAAH polyclonal antiserum. FAAH was identified as a predicted 70 kDa protein expressed at constant levels throughout all the different stages of Dictyostelium development suggesting an essential role for FAAH throughout development. However, expression levels of *in vivo* FAAH protein in Dictyostelium wild type cells were very low and several attempts to study protein localization by cell fractionation and Western blotting were not successful. The inability to detect endogenous FAAH protein in the fractionation experiments may be due to very low level of protein expression or due to protein getting degraded during the process of fractionation. Therefore, AX3FAAH cells were used in cell fractionation studies. Cells grown in liquid nutrient were harvested and fractionated into membrane and cytosol fractions by differential centrifugation. At 13,000*xg*, FAAH was distributed in both pellet and supernatant fractions (Figure 6) indicating that FAAH may be a plasma membrane associated protein. At 100,000*xg*, FAAH was predominantly present in pellet fraction further indicating that FAAH may be associated with other intra cellular membrane bound organelles. The small quantities of FAAH in the supernatant after this spin strongly suggest a predominantly membrane associated protein and is further supported by increased yields of HIS-FAAH when detergents such as Triton X-100 are added. Unlike other mammalian FAAHs, Dictyostelium FAAH does not have any predicted transmembrane domain. Similar membrane associated behaviour was reported when human FAAH was expressed as a recombinant protein lacking a N-terminal transmembrane domain and the protein was predominantly present in membrane fractions [23].

## Discussion

Bioinformatics analysis of FAAH amino acid sequence revealed the presence of an amidase signature domain, which is similar to that present in other mammalian FAAH. The amidase signature sequence is conserved among many proteins from the amidase class, which include enzymes hydrolyzing acetamide, acrylamide, nicotinamide, and glutamide [24-27]. FAAH is the only characterized mammalian enzyme belonging to the amidase class and recently the FAAH homolog from Arabidopsis has been characterized and reported to belong to the amidase class.

Despite Dictyostelium FAAH’s considerable deviations in sequence identity across full length amino acid sequences when compared to human, porcine, rat and Arabidopsis sequences, Dictyostelium FAAH has retained anandamide hydrolysis function. Recombinant FAAH produced from Dictyostelium and *E.coli* was capable of hydrolyzing anandamide and other fatty acid substrates arachidonoyl *p*-nitroaniline and decanoyl *p*-nitroaniline similar to other characterized FAAHs. Previously, Schmid and co-workers reported *N*-acylethanolamine amidohydrolase from rat liver which hydrolyzed various *N*-acylethanolamines [28] but did not test anandamide as a substrate. Later when Cravatt’s group cloned and characterised *N*-acylethanolamine amidohydrolase cDNA, the enzyme hydrolysed anandamide in addition to other fatty acid amides. These findings indicated that the enzyme may regulate growing family of bioactive fatty acid amides, and the enzyme was renamed as fatty acid amide hydrolase.

Kinetic parameters indicate that Dictyostelium FAAH has preferred affinity for longer unsaturated acyl chains and inhibition by PMSF, LY2183240 and MAFP suggest a conserved enzyme mechanism between Dictyostelium and mammalian FAAH [29,30]. These preliminary biochemical and kinetic analyses of Dictyostelium FAAH supports the identification of [GenBank: XM_638290] as a functional homolog of mammalian FAAH. *N*-acylphosphatidylethanolamines (NAPEs) and its hydrolysed product *N*-acylethanolamines (NAEs) have been previously reported in Dictyostelium [31]. Identification of FAAH in Dictyostelium indicates FAAH may be a potential regulator of NAEs produced in Dictyostelium cells. Among many established physiological roles for anandamide in mammalian cells, recently a role in neutrophil chemotaxis was identified [32] and therefore we predict a similar kind of role for NAEs that may exist in Dictyostelium. As recent advances are made to develop FAAH inhibitors for potential novel therapeutics, having a mammalian FAAH homolog in Dictyostelium should offer an additional and moreover simple eukaryotic model system to screen any relevant drugs for their pharmacological influence at the molecular and cellular level.

## Conclusions

Our study indicates that Dictyostelium produces anandamide hydrolysing enzyme throughout its development life cycle. This is the first report on the identification of anandamide hydrolyzing enzyme in Dictyostelium, suggesting the potential of Dictyostelium as a simple eukaryotic model system to study the mechanisms of action of any FAAH inhibitors as drug targets.

## Methods

Anandamide, arachidonoyl *p*-nitroaniline, decanoyl *p*-nitroaniline and methyl arachidonoyl fluorophosphonate (MAFP) were purchased from Cayman Chemicals (Ann Arbor, MI, USA). Phenylmethylsulfonyl fluoride (PMSF) Dimethyl sulfoxide (DMSO), isopropyl-1-thio-β-D-galactopyranoside (IPTG), *p*-nitroaniline, and Freund’s complete and incomplete adjuvant were purchased from Sigma-Aldrich Canada (Oakville, ON, Canada). All media were obtained from Difco Laboratories (Detroit, MI). All restriction endonucleases were obtained from New England Biolabs (Mississauga, ON, Canada). T4 DNA ligase, Taq polymerase and G418 were purchased from Invitrogen (Burlington, Ontario, Canada). PCR amplification reactions were performed with a GeneAmp PCR system 9700 thermocycler (Applied Biosystems Canada, Streetsville, ON, Canada). PWO polymerase was purchased from Roche Applied Science (Laval, Quebec, Canada).

### Dictyostelium strain growth and development

*Dictyostelium discoideum* AX3 cells were grown either with *Klebsiella aerogenes* on SM agar plates or in Sorensen’s phosphate buffer (2 mM Na_2_HPO_4_, 14.6 mM KH_2_PO_4_, pH 6.0). Cells were grown axenically in liquid nutrient medium [33] with shaking of the suspension at 150 rpm at 22-24°C. AX3FAAH cells were cultured in axenic liquid nutrient medium containing 10 μg ml^-1^ G418 for selection of the recombinant protein producing cells. To analyze development, cells were grown axenically to a density of 2–3 × 10^6^ cells ml^-1^ washed twice in Sorensen’s phosphate buffer and 5 × 10^7^ cells were plated on phosphate agar plates**.** At different time points during development cells were harvested and total proteins extracted. Cell density was determined by taking an aliquot of the culture and counting it in a standard hemocytometer.

### Dictyostelium subcellular fractionation

For separation of membrane and cytosolic fractions, cells were washed in Sorensen’s phosphate buffer and resuspended at a density of 1 × 10^8^ cells ml^-1^ in MES buffer (20 mM MES, pH6.5, 1 mM EDTA, 250 mM sucrose) supplemented with complete protease inhibitor mixture, EDTA-free (Roche Applied Science, Laval, Quebec, Canada). Cells were lysed by sonication, membrane and cytosolic fractions were separated by two separate centrifugation forces at 15,000*xg* and 100,000*xg* for 30 min at 4°C. Complete lysis of the cells after sonication was confirmed by checking for no intact cells under the microscope.

### Bioinformatics and cDNA isolation

Nucleotide BLAST searches (http://dictybase.org/tools/blast) were performed using full length human FAAH nucleotide sequences. Dictyostelium DNA sequences coding for characteristic amidase signature (AS) motifs were identified in the annotated genome data base (http://dictybase.org) and ortholog *DDB_G0275967* (http://dictybase.org/gene) [GenBank: XM_638290] was selected for further functional characterization. Domain architecture analyses and amino acid sequence homology comparisons among FAAH from different species were done using sequence analysis tools available at http://www.ncbi.nlm.nih.gov/guide/sequence-analysis/ and http://www.ebi.ac.uk/Tools/clustalw2/index.html. Based on gene exon sequence information of [GenBank: XM_638290], oligonucleotides were designed and used in reverse transcription-polymerase chain reaction (RT-PCR) for complete cDNA synthesis. Total RNA was extracted using RNeasy Midi kit (Qiagen, Mississauga, Ontario, Canada) from vegetatively grown Dictyostelium cells according to manufacturer’s instruction. 2μg of RNA was used in the RT reaction using Omniscript RT Kit (Qiagen, Mississauga, Ontario, Canada), 100 pmol of the gene specific primer NRC 190 with sequence 5’GTCGACTTAGTTATTTGGGTTTGTGCAATTTG 3’ and 100 pmol of Oligo-dT primer (Qiagen) was used in the RT reaction according to manufacturer’s instructions. The cDNA obtained was used as the template in the subsequent polymerase chain reaction (PCR) to amplify the FAAH gene using gene specific primers NRC189 with sequence 5’CATATGCACCACCATCATCACCACACATCTTCTTCATTAAGTAAAAGTAGTAG3’ and NRC 190. Primer NRC189 contained a restriction enzyme *NdeI* site and nucleotides coding for 6 histidine (HIS) residues and primer NRC190 contained a restriction enzyme *SalI* site. PCR cycle conditions were 94°C melting (1 min), 55°C annealing (1 min), and 68°C extension (2.0 min), and after 20 cycles of amplification, the PCR product obtained was ligated into pCR2.1 plasmid using TA cloning kit (Invitrogen, Burlington, Ontario, Canada) according to manufacturer’s instruction. The ligated FAAH cDNA in pCR2.1 was transferred by electroporation into *E.coli* TOP10F’ (Invitrogen). The clones obtained were examined by sequencing using M13 forward and reverse primers for having the correct cDNA insert and the right clone was called as pCR2.1-FAAH.

### Cloning of FAAH into HIS tag fusion protein expression system in Dictyostelium

FAAH was expressed as a tagged protein, fused with 6 Histidine (HIS) residues at the N-terminal end of FAAH using the pDEXRH expression vector [34]. Two oligonucleotides were synthesized for use in the PCR amplification of FAAH cDNA from the vector pCR2.1-FAAH containing full length FAAH cDNA. Oligonucleotides NRC214 with sequence 5’AAGCTTAAAAAATGCACCACCATCATCACCACACATCTTCTTCATTAAGTAAAAGTAGTAG3’and NRC215 with sequence 5’AAGCTTTTAGTTATTTGGGTTTGTGCAATTTG3’ were used as 5’ and 3’ primers respectively. Primer NRC214 contained a *HindIII* restriction enzyme site and nucleotides coding for 6 histidine (HIS) residues and primer NRC215 contained a *HindIII* restriction enzyme site that allowed insertion of the PCR fragment into pDEXRH vector. PCR cycle conditions were 94°C melting (1 min), 54°C annealing (1 min), and 68°C extension (2.0 min), and after 20 cycles yielded sufficient DNA to proceed with the cloning steps. The PCR product obtained was digested with restriction enzyme *HindIII* and ligated into *HindIII* digested pDEXRH vector. The ligated FAAH cDNA was transferred into *E.coli* DH10B by electroporation. The clones obtained were examined for having the full length FAAH cDNA insert by restriction digestion mapping and DNA sequencing using gene specific primers. The right clones obtained in *E.coli* DH10B were designated pDEXRH-FAAH. The protein expression plasmid pDEXRH-FAAH was transformed into Dictyostelium strain AX3 by electroporation [35] with the Gene pulser XCell (Bio-Rad). The Dictyostelium target strain was screened by selecting on G418 antibiotic for cells that produced a 70 kDa fusion protein. The Dictyostelium cell line which expressed HIS-FAAH fusion protein was designated AX3FAAH.

### Expression of HIS-FAAH protein and purification using nickel–nitrilotriacetic acid resin (Ni-NTA) from Dictyostelium

A 20 ml culture of Dictyostelium expression strain AX3FAAH at a density of 3x10^6^ cells ml^-1^ was inoculated into 1 L of liquid nutrient medium in a 4 L Erlenmeyer flask and shaken at 150 rpm at 22-24°C. Cell density was determined by taking an aliquot of the culture and counting it in a standard hemocytometer. For all the AX3FAAH expression cultures, G418 antibiotic at a concentration 10 μg ml^-1^ was added to maintain the selection pressure on the integrated recombinant plasmid. When the culture reached a cell density of 3x10^6^cells ml^-1^, the cells were harvested and pelleted at 1000*xg* for 10 min at 4°C. The cells were lysed by freeze thaw using lysis buffer (20 mM Tris-Cl, 200 mM KCl, 10 mM Imidazole,10 mM *β*-mercaptoethanol and 0.1% Triton X-100, pH 9.0) containing complete protease inhibitor mixture, EDTA-free (Roche Applied Science, Laval, Quebec, Canada) and homogenized using a Wheaton Potter-Elvehjem homogenizer with a PTFE pestle (Fisher Scientific). Homogenized lysates were centrifuged at 100,000*xg* for 50 min at 4°C, and the supernatant fraction was batch bound to 3 ml of Ni-NTA resin (Qiagen) at 4°C for 1 h. Protein bound Ni-NTA resin was then packed in a column using gravity flow. The column was washed with 10 column volumes of lysis buffer containing10mM imidazole and 300 mM KCl. To elute the protein of interest a linear gradient was applied from 10 to 100 mM imidazole in lysis buffer over 30 column volumes before a final pulse of 10 column volumes of lysis buffer containing 200 mM imidazole. Fractions containing the purified protein of interest as determined by SDS–PAGE (10%) and Coomassie staining were pooled and dialyzed overnight against dialysis buffer (20 mM Tris-Cl, pH 9.0, 50 mM NaCl) at 4°C. Protein concentrations were estimated by Bradford assay and the yields were typically 2–4 mg L^–1^ of cell culture.

### Cloning of FAAH into maltose binding protein (MBP) fusion expression system in *E.coli*

FAAH was expressed as a tagged protein, fused with maltose binding protein using pCWMalET expression vector [36]. Full length FAAH cDNA containing a HIS tag at the N-terminus was obtained by digesting pCR2.1-FAAH plasmid with restriction enzymes *NdeI* and *SalI* and ligated into *NdeI* and *SalI* digested pCWMalET vector and the clone obtained was designated pCWMalET-FAAH. The clone obtained was examined for protein expression in *E.coli* BL21 [DE3] (Novagen, Madison, WI).

### Expression of MBP-FAAH fusion protein and purification using amylose resin

A fresh overnight culture of BL21 containing pCWMalET-FAAH vector was diluted 100 fold in LB medium containing 100μg/ml of ampicillin. 1 to 4 liters of culture was grown at 25°C in the presence of 0.2% glucose, induced at an OD600 of 0.6 with 0.1 mM isopropyl-1-thio-β-D-galactopyranoside and harvested 5 h later. Cell pellets were resuspended in lysis buffer (20 mM Tris-Cl, pH 9.0, 200 mM NaCl, 1 mM EDTA, 10 mM-*β*-mercaptoethanol) containing complete protease inhibitor mixture, EDTA-free (Roche Applied Science). The cells were disrupted by two passes through an emulsiflex C5 (20,000 psi) (Avestin, Ottawa, Canada). Lysates were centrifuged at 100,000*xg* for 50 min at 4°C, and the supernatant fraction was batch bound to 3 ml of amylose resin (NEB, Pickering, Ontario) at 4°C for 1 h. Protein bound amylose resin was then packed in a column using gravity flow. The column was washed with 10 column volumes of lysis buffer containing 300 mM NaCl and the Protein of interest was eluted using 15 mM maltose in lysis buffer over 5 column volumes. Fractions containing purified protein of interest were determined by sodium dodecyl sulfate–polyacrylamide gel electrophoresis (SDS–PAGE) (10%) and Coomassie staining and pure protein fractions of interest were pooled and dialyzed overnight against dialysis buffer (20 mM Tris-Cl, pH 9.0, 50 mM NaCl) at 4°C. Yields of purified protein were typically 8–10 mg L^–1^ of cell culture.

### FAAH Enzyme activity assays

Hydrolysis of anandamide by HIS-FAAH and MBP-FAAH was determined by Capillary electrophoresis electron spray mass spectroscopy (CE-ES-MS). To 100μl of reaction buffer (20 mM Tris–HCl, pH 9.0, 50 mM NaCl, 10% DMSO) was added 100μg of anandamide substrate from 10 mg ml^-1^ stock prepared in methyl acetate. Enzyme reaction was initiated by adding 10μg of HIS-FAAH or MBP-FAAH enzyme (from 0.2 mg ml^-1^ stock, in 20 mM Tris-Cl, pH 9.0, 50 mM NaCl) and incubated at 37°C for 30 min and the reaction was analyzed by CE-ES-MS. For kinetic analysis, the rates of FAAH catalyzed hydrolysis of *p*-nitroanilide substrates, arachidonoyl *p*-nitroaniline (A*p*NA) and decanoyl *p*-nitroaniline (D*p*NA) were determined by monitoring the release of *p*-nitroaniline (*ϵ =*13500 M^-1^ cm^-1^) at 380 nm using a microplate reader (PowerWave X, Biotech Instruments Inc., Winooski, VT.). Substrate conversion was extrapolated from *A*_380_ versus *p*-nitroaniline standard curves using microplate reader. Specifically, enzyme reactions were initiated by adding 50μl HIS-FAAH enzyme (from 0.2 mg ml^-1^ stock, in 20 mM Tris-Cl, pH 9.0, 50 mM NaCl) to 100μl of reaction buffer (20 mM Tris-Cl, pH 9.0, 50 mM NaCl and 0.5% Triton X-100,) containing different concentration (20-300μM) of A*p*NA and D*p*NA substrates made in DMSO and the final concentration of DMSO in the reaction was adjusted to 10%. Enzyme reactions were performed in 96-well microplate at 37°C, presence of 0.5% Triton X-100 and 10% DMSO. Enzyme specific activity points were determined in triplicate and fitted into Michaelis-Menten curve.

### Capillary electrophoresis electrospray mass spectroscopy (CE-ES-MS) analysis of anandamide hydrolysis by FAAH

CE-ES-MS analyses of enzymatic reactions were performed on an Applied Biosystems/MDS Sciex 4000QTRAP (Concord, ON, Canada) coupled to a Prince Technologies CE system (Emmen, The Netherlands), using chloroform-methanol (50%v/v) as the separation buffer. Spectra were acquired using precursor ion scanning for anandamide (*m/z* 346.3) and its hydrolyzed product arachidonic acid (*m/z* 303.5) using negative-ion mode, with orifice voltage and electron spray needle voltage set at 30 V and −5.4 kV respectively.

### Production of anti FAAH polyclonal antibody

Polyclonal antibodies specific for FAAH were obtained by immunizing New Zealand white rabbits with recombinant FAAH protein purified from *E.coli*. Recombinant MBP-FAAH protein expressed in *E.coli* was purified using amylose resin and pure MBP-FAAH was cleaved by thrombin, to separate FAAH from MBP. Pure FAAH was obtained by Ni-NTA affinity purification and was used to immunize two rabbits using 100μg of protein antigen per animal. At day 1 prime immunization of 100μg ml^-1^ of antigen was done using complete Freund’s adjuvant (according to manufacturer’s instruction) by sub-cutaneous injection of 0.5 ml at two sites. At day 28 animals were boosted with 100μg ml^-1^ protein per animal using incomplete Freund’s adjuvant. At day 56 a second booster injection identical to the first booster injection was performed and at day 69 the animals were bled to check for the antibody titre.

### Gel electrophoresis and Western blotting

Protein samples diluted with 1:1 sample buffer (60 mM Tris–HCl, pH 6.8, 2% SDS, 10% glycerol, 0.025% bromophenol blue) were separated on 10% polyacrylamide – SDS gels. For Western blotting analysis, separated proteins were electrophoretically transferred onto a polyvinylidene fluoride membrane (PVDF, 0.2μm, BioRad). Protein bound PVDF membranes were blocked with 5% milk and incubated with polyclonal anti-FAAH antibody raised in rabbits at a dilution of 1:2000 and secondary antibody anti-rabbit IgG conjugated to horseradish peroxidase (Sigma-1:3000) to detect FAAH from wild type cells. To detect HIS tagged recombinant proteins PVDF membrane were incubated with horseradish peroxidase (HRP) conjugated anti-HIS antibody (Sigma- 1:3000) and analyzed using Western Pico chemiluminescence (Pierce) and X-ray film exposure.

## Abbreviations

NAEs, N-acylethanolamines; AS, Amidase signature; FAAH, Fatty acid amide hydrolase; MAFP, Methyl arachidonoyl fluorophosphonate; PMSF, Phenylmethylsulfonyl fluoride; ApNA, Arachidonoyl p-nitroaniline; DpNA, Decanoyl p-nitroaniline; MBP, Maltose binding protein; DMSO, Dimethyl sulphoxide.

## Competing interests

None of the authors have any competing interests to declare.

## Authors’ contributions

DN designed and executed all the experiments, and drafted the manuscript. ICS helped in enzyme kinetics data analysis. JCR and ADC supervised the work and revised the manuscript. All authors read and approved the final manuscript.
